# Epigenetic Mechanisms Underlying Sex Differences in Neurodegenerative Diseases

**DOI:** 10.3390/biology14010098

**Published:** 2025-01-19

**Authors:** Andrea Stoccoro

**Affiliations:** Laboratory of Medical Genetics, Department of Translational Research and of New Surgical and Medical Technologies, Medical School, University of Pisa, Via Roma 55, 56126 Pisa, Italy; andrea.stoccoro@unipi.it

**Keywords:** epigenetics, sex bias, neurodegenerative diseases, Alzheimer’s disease, Parkinson’s disease, amyotrophic lateral sclerosis

## Abstract

Neurodegenerative diseases are characterized by profound differences between females and males in terms of incidence, clinical presentation, and disease progression. Furthermore, there is evidence suggesting that differences in sensitivity to medical treatments may exist between the two sexes. Although the role of sex hormones and sex chromosomes in driving differential susceptibility to these diseases is well-established, the molecular alterations underlying these differences remain poorly understood. Epigenetic mechanisms, including DNA methylation, histone tail modifications, and the activity of non-coding RNAs, are strongly implicated in the pathogenesis of neurodegenerative diseases. While it is known that epigenetic mechanisms play a crucial role in sexual differentiation and that distinct epigenetic patterns characterize females and males, sex-specific epigenetic patterns have been largely overlooked in studies aiming to identify epigenetic alterations associated with neurodegenerative diseases. This review aims to provide an overview of sex differences in epigenetic mechanisms, the role of sex-specific epigenetic processes in the central nervous system, and the main evidence of sex-specific epigenetic alterations in three neurodegenerative diseases, including Alzheimer’s disease, Parkinson’s disease, and amyotrophic lateral sclerosis. Understanding the sex-related differences of these diseases is essential for developing personalized treatments and interventions that account for the unique epigenetic landscapes of each sex.

## Simple Summary

Neurodegenerative diseases are complex disorders that, in most cases, arise from the interaction between genetic susceptibility and environmental risk factors. In this context, epigenetic mechanisms are crucial in neurodegenerative diseases’ pathogenesis, mediating environmental factors’ effects on proper genome functionality. Sex is an important variable in modulating the risk of developing neurodegenerative diseases and influences their clinical course. Although it is well established that epigenetic mechanisms are also influenced by sex, epigenetic differences between females and males affected by neurodegenerative diseases remain poorly investigated. This review summarizes the key evidence of sex-specific epigenetic alterations in major neurodegenerative diseases, including Alzheimer’s disease, Parkinson’s disease, and amyotrophic lateral sclerosis. Given the growing importance of gender medicine in the treatment of human diseases, a deeper understanding of sex-specific epigenetic mechanisms is highly relevant for neurodegenerative diseases.

## 1. Introduction

The increasing number of individuals affected by neurodegenerative diseases is exerting a significant and escalating strain on public healthcare systems and social welfare worldwide. This trend is largely driven by medical advancements that have increased life expectancy. Currently, approximately fifty million people globally are affected by Alzheimer’s disease (AD) or other types of dementia, a number expected to triple by 2050 [1]. Similarly, the number of people diagnosed with Parkinson’s disease (PD) has increased twofold in the last thirty years and is anticipated to grow by the same magnitude over the next twenty years [2]. Likewise, the number of people affected by motor neuron diseases, such as amyotrophic lateral sclerosis (ALS), has risen by approximately 12.4% between 1990 and 2019 [3]. As a result, the need to identify effective treatments for these human pathologies or to identify procedures to hinder or postpone the symptoms’ onset has become increasingly critical.

In recent years, epidemiological and clinical studies have drawn attention to the influence of biological sex on neurodegenerative diseases, as several differences between females and males in the incidence and clinical course of these disorders are known [4]. For example, approximately two-thirds of AD patients are women [5]. Moreover, women tend to experience a more accelerated decline in cognitive and physical functions, and also have a longer disease duration and a higher prevalence of concomitant neuropsychiatric disorders [6,7]. The primary explanation proposed for the higher prevalence of AD in women is their greater average lifespan compared to men. However, research examining the age-specific incidence rates of AD and other dementias between men and women has produced conflicting results [5]. In this context, PD is also marked by sex differences in disease risk and progression. Unlike AD, however, males have a significantly higher risk, up to twice that of females, of developing PD. However, women with PD often exhibit a higher mortality rate and faster disease progression [8]. Additionally, there are notable sex-based differences in motor and nonmotor symptoms, treatment responses, and disease risk factors. For instance, women are more likely to develop tremor-dominant PD but tend to have less rigidity than men and show greater improvement in daily living activities following deep brain stimulation [9]. Distinct sex-specific characteristics have also been reported in ALS patients. Males are more often affected than females and usually exhibit an earlier onset of symptomatology. Differences between sexes also exist regarding the clinical features of ALS, with a greater proportion of female patients with bulbar onset and a greater proportion of male patients with spinal onset [10].

Overall, sex-related differences in neurodegenerative diseases suggest that disease development may involve distinct pathogenic mechanisms in males and females, or that the same mechanisms may operate differently between the sexes. A deeper understanding of the sex-specific mechanisms underlying neurodegenerative diseases is essential for identifying sex-specific biomarkers for diagnosis and prognosis, and for developing more targeted interventions and treatments aimed at delaying disease onset and improving clinical outcomes. Several intrinsic biological risk factors are gaining importance in supporting sex differences in the risk of developing neurodegenerative diseases. Among these factors, sexual dimorphism in central nervous system structures, alterations in sex hormone signaling, sex-specific risk genes, immune responses, and vascular disease are considered significant determinants [11,12].

However, molecular mechanisms underlying sex differences in neurodegenerative diseases remain elusive. In recent years, evidence of the pivotal role of epigenetics in the etiopathogenesis of neurodegenerative diseases has exponentially increased. Epigenetic mechanisms, which regulate gene expression and chromatin conformation, are highly sensitive to environmental factors. As a result, they play a crucial role in the pathological processes underlying neurodegeneration [13]. Interestingly, epigenetic mechanisms have also been proposed as pivotal mediators of sex-related differences in human diseases, including autoimmune disorders, various types of cancers, and infectious diseases [14]. However, the role of sex differences in epigenetic mechanisms in neurodegenerative diseases has been greatly overlooked. This review aims to address the potential role of sex-related epigenetic mechanisms in neurodegenerative diseases. Sex differences in epigenetic mechanisms are first shown, followed by an overview of sex-specific epigenetic modifications important for brain development and normal adult brain physiology. Finally, the key evidence available in the literature up to December 2024 regarding sex-related epigenetic alterations in three neurodegenerative diseases, namely, AD, PD, and ALS, is described.

## 2. Epigenetic Mechanisms: Differences Between Sexes

### 2.1. Overview of the Main Epigenetic Mechanisms

**Figure 1:**
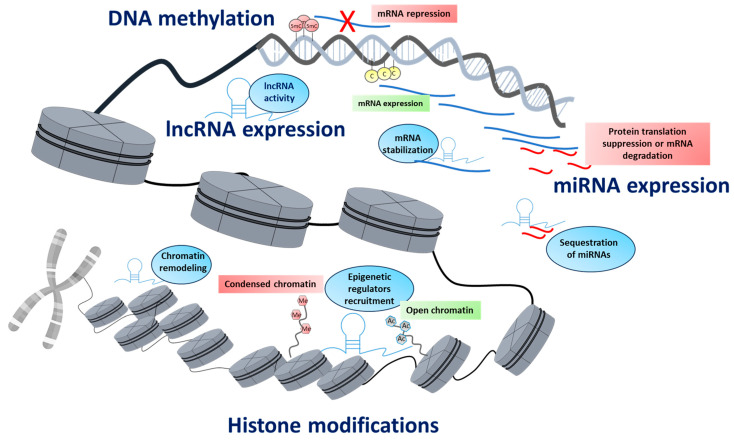
Overview of the main epigenetic mechanisms. The methylation of CpG sites (5mC) is usually associated with the repression of gene expression, while demethylated cytosine residues (C) allow mRNA expression. LncRNAs exert their epigenetic activity through several pathways (indicated in blue circles). The binding of miRNAs to their target mRNAs suppresses protein translation or induces mRNA degradation. Histone modifications regulate chromatin accessibility by adding or removing molecules from histone tails. For example, the trimethylation of histone H3 at lysine 9 (H3K9me) promotes a condensed chromatin state, while histone acetylation (Ac) facilitates an open chromatin configuration.

The term epigenetics refers to the study of inheritable gene expression changes that occur in the absence of DNA sequence modifications. These changes are primarily regulated by three main mechanisms, namely, DNA methylation, histone tail modifications, and non-coding RNA activity (Figure 1) [15]. DNA methylation consists of the addition of a methyl group to the DNA, usually at the level of a cytosine followed by a guanine residue, the CpG site, forming 5-methylcytosine (5-mC). Usually, DNA methylation is associated with gene expression repression, especially when it occurs in gene promoters, but it can also be associated with activated gene expression, especially when it occurs in the gene body [16]. Histone modifications involve the addition or removal of molecules at the N-terminal tails of histone proteins. The main characterized histone modifications include acetylation, methylation, phosphorylation, and ubiquitination, among others [17]. The addition of these molecules to the histones modulates the interaction between the histones and the DNA molecule, enabling a more relaxed chromatin conformation, e.g., following histone acetylation, or a more compacted chromatin, e.g., methylation of residues lysine 27, which are associated with activated and repressed gene expressions, respectively. Various enzymes regulate epigenetic mechanisms and are classified based on their function as writers, erasers, and readers. Writers introduce epigenetic marks, as exemplified by DNA methyltransferases (DNMTs) and histone acetyltransferases (HATs). Erasers, on the other hand, delete these epigenetic marks, such as the removal of acetyl groups from histone tails by histone deacetylases (HDACs). Readers, such as the methyl-CpG-binding domain (MBD) proteins, are involved in the detection of and binding to epigenetic marks, facilitating functional outcomes by recruiting other proteins or complexes [18]. A further layer of epigenetic regulation of gene expression and chromatin conformation is mediated by non-coding RNAs (ncRNAs). Based on their length, ncRNAs are usually classified as short RNAs, such as microRNAs (miRNAs), which are composed of 22–25 nucleotides, and long non-coding RNAs (lncRNA), which have more than 200 nucleotides [19]. MiRNAs regulate gene expression post-transcriptionally by binding to mRNA untranslated regions (UTRs), typically within the 3′-UTR, to suppress protein translation or promote mRNA decay [20]. LncRNAs, through RNA-RNA, RNA-DNA, and RNA-protein interactions, have several biological functions, including the modification of chromatin conformation, the regulation of gene transcription, splicing RNA, protein translation and localization, and other forms of RNA processing [21].

### 2.2. Sex-Related Differences of Epigenetic Mechanisms

Beyond the regulation of gene expression, epigenetic mechanisms are involved in a plethora of molecular pathways that are pivotal for sexual differentiation. For example, it has been reported that DNA methylation and histone modifications are involved in the expression of the Sex-determining Region Y (*Sry*), a gene located on the Y chromosome that encodes for a transcription factor essential for male gonadogenesis [22]. Studies in mice have shown that the *Sry* locus, which is rich in CpG sites, is highly methylated during the early postnatal days when the gene is not expressed. In contrast, it becomes hypomethylated in the gonads during the later postnatal period, coinciding with the onset of *Sry* expression for gonadal differentiation [23]. Similarly, the expression of the *KDM3A* gene, which encodes a histone demethylase specifically targeting the repressive H3K9me2 histone modification, has been reported to be correlated with the expression pattern of *Sry* and mutant mice with *KDM3A*-deficiency, showing male-to-female sex reversal [24,25]. It has also been suggested that *Sry* itself may act as a regulator of epigenetic mechanisms, influencing gene transcription through the modulation of DNA methylation, histone tail modifications, and miRNA expression [26]. For instance, *Sry* has been shown to regulate the expression of *miR-138*, which is involved in various pathways, including the regulation of short-term memory and the inhibition of synaptic transmission [27,28]. Moreover, epigenetic mechanisms are involved in X inactivation (XCI), the process through which one of the two X chromosomes in female cells is inactivated. The XCI process is initiated by the lncRNA *XIST* (X-inactive Specific Transcript), which is transcribed from the X-inactivation center on the X chromosome destined for inactivation. *XIST* coats the X chromosome and recruits various protein complexes, including HDACs, histone methyltransferases, and DNMTs, driving significant changes in chromatin organization [29]. As a result of the XCI process, the inactivated X chromosome adopts a compact heterochromatic state so that females will have only one functional copy of the X chromosome in each cell, although several X-linked genes are known to escape X-inactivation, leading to a doubled dosage of these genes in XX cells relative to XY cells [30]. The importance of a sex chromosome pattern and escape genes in influencing disease susceptibility is highlighted by studies on individuals with sex chromosome aneuploidies, such as Klinefelter syndrome (XXY) and Turner syndrome (X0) [31]. For instance, autoimmune diseases typically affect females more frequently than males, with female-to-male ratios as high as 9:1 in Sjögren’s syndrome and 7:1 in systemic lupus erythematosus [32]. Notably, males with two X chromosomes (Klinefelter syndrome) exhibit a higher prevalence of Sjögren’s syndrome compared to control males [33], whereas females with a single X chromosome (Turner syndrome) show reduced susceptibility to systemic lupus erythematosus [34].

Thus, epigenetic differences between females and males emerge early during development. Multiple studies have reported that inherent differences in epigenetic mechanisms also exist between males and females during adulthood. DNA methylation signatures of autosomes specific for females and males have been frequently identified in peripheral blood [35,36,37,38,39,40] and have also been reported in the liver [41], adipose tissue [42], and skeletal muscle [43]. Similarly, sex-specific DNA methylation signatures have been reported in developing [44,45] and adult human brains [46], indicating that most sex differences in the brain methylome manifest early in fetal development and are stable across the life course. Of note, a meta-analysis performed on DNA methylation data at the genome-wide level of 7333 peripheral blood and tissue samples, of which 3647 were from female subjects, identified 184 autosomal CpG sites differentially methylated between females and males [47]. Specifically, males showed a slight, but significant, increase in global DNA methylation. Interestingly, the top associated CpG sites were in genes involved in sex-specific functions, such as *SLC9A2* and *NUPL1*, which are important for male infertility, and *DDX43*, which is involved in spermatogenesis [47]. Moreover, it has been reported that several CpG sites showing sex-related DNA methylation patterns undergo age-related modifications, and in several instances, these male–female differences in methylation are largely maintained throughout aging. For example, Yusipov et al. reported that a large fraction (43%) of sex-associated CpG sites undergo age-associated DNA methylation changes in peripheral blood, and eight CpG sites also showed an age-by-sex interaction [48]. Similarly, a DNA methylation study conducted on peripheral blood from over 2500 individuals aged 18 to 87 years, along with a replication cohort of more than 4400 individuals aged 18 to 93 years, identified multiple autosomal and X-linked CpG sites associated with 251 genes that exhibited an age-by-sex interaction [49]. The CpG dinucleotide showing the most significant difference in DNA methylation levels between sexes was located in the X-linked *GAGE10* gene. Particularly, DNA methylation levels of *GAGE10* remained stable over time in males but declined with age in females. In recent years, many studies have identified DNA methylation changes related to age at individual CpG sites, contributing to the evolution of predictive algorithms known as “epigenetic clocks”, which estimate the biological age based on epigenetic profiles [50]. While a definitive link between epigenetic clock predictions and the risk of age-related diseases remains elusive [51], numerous independent studies have demonstrated that epigenetic age acceleration, defined as a predicted epigenetic age exceeding chronological age, is correlated with age-associated conditions such as cancer, cardiovascular diseases, neurodegenerative disorders, and increased all-cause mortality [52]. Conversely, epigenetic age deceleration has been associated with successful aging and increased longevity [53,54]. Interestingly, differences in epigenetic age acceleration between sexes have been reported. For example, it was shown that men have higher epigenetic aging rates than women in blood, saliva, and brain tissue [55]. More recently, the epigenetic clock was assessed in a Finnish population consisting of young twins (21–42 years), older twins (50–76 years), and 151 pairs of young (21–30 years) opposite-sex twins [56]. The authors observed that men were biologically older than women, and the association between sex and biological aging was stronger in older twins. Similarly, Swedish women exhibited lower DNA methylation ages than men [57]. The difference in biological age between females and males could be partially explained by hormonal factors. Indeed, it has been reported that menopause is associated with an accelerated biological age [58], while androgen reduction is associated with a decelerated biological age [59].

Also, histone modifications have been reported to differ between males and females. For example, levels of acetylated (H3K9/14Ac) and trimethylated (H3K9Me3) histone H3 were found to be increased in the cortex and hippocampus of male mice compared to females on embryonic day 18, the day of birth, and six days later [60]. Moreover, analysis at the genomic level of the histone-3 lysine-4 trimethylation (H3K4me3) in the stria terminalis and preoptic area of female and male mice showed that females had a greater distribution of the epigenetic mark compared to males [61]. Similarly, in addition to the already described lncRNAs *XIST*, several ncRNAs have been reported to be expressed differently between males and females. A total of 375 lncRNAs were differentially expressed in mouse liver between females and males, including 123 male- and 252 female-biased lncRNAs [62]. A recent study investigated the sex differences in constitutive long non-coding RNA expression in spinal cord and skeletal muscle from wild-type mice [63]. Authors found age- and tissue-dependent significant sex differences, being more prominent in skeletal muscle. Moreover, in vitro analysis showed that long non-coding RNA levels are modulated by estradiol and dihydrotestosterone treatment in muscle tissue [63]. Moreover, some evidence suggests that escape genes from XCI can contribute to sexual dimorphism. For example, the escape gene encoding for the miR-548am-5p confers to XX cells a higher resistance to cell death compared to XY cells, a characteristic that could favor the survival of the female organism [64].

Epigenetic mechanisms are sensitive to hormonal effects, and sex hormones, including androgens and estrogens, may play a crucial role in establishing epigenetic pattern differences between females and males. Sex hormones are steroid hormones that can enter the cell, where they bind to specific receptors, such as an estrogen receptor (ER) and androgen receptor (AR) [65]. Once activated, the hormone–receptor complex enters the nucleus, dimerizes, and binds to specific hormone response elements on DNA to regulate transcription of target genes [66]. However, there is also evidence that sex hormones regulate gene expression through their modulation of epigenetic mechanisms. For example, the expression of *DNMT1*, *DNMT3A*, and *DNMT3B* is regulated by female sex steroid hormones during the menstrual cycle [67]. Additionally, estradiol, derived from testosterone conversion, was shown to elevate *DNMT3A* and *DNMT3B* expression [68]. Recently, it has been reported that *DNMT1*, *DNMT3A*, and *DNMT3B* genes have several binding sites for estrogen hormone binding, thus further suggesting that their expression is strongly regulated by sex hormones [69]. Moreover, sex hormones are involved in the modulation of genes encoding for writers and erasers of histone tail acetylation and methylation [70,71]. Thus, sex hormones are key regulators of epigenetic mechanisms and may underlie the epigenetic differences between females and males, contributing to their differing susceptibility to certain diseases.

Also, various environmental factors play a significant role in regulating epigenetic mechanisms [72]. Indeed, diet, stress, air pollution, and chemicals like endocrine disruptors can cause lasting epigenetic changes that affect sexual differentiation, development, and aging [73]. Epigenetic mechanisms are particularly vulnerable to environmental influences during fetal development, potentially predisposing individuals to complex diseases later in life [74]. The Developmental Origins of Health and Disease (DOHaD) theory posits that early-life exposure to adverse environmental factors heightens the risk of adult-onset complex diseases, including neurodegenerative disorders, with epigenetic mechanisms as key mediators of this process [74]. For instance, animal studies have demonstrated that in utero exposure to heavy metals and pesticides increases the risk of AD and PD through the alteration of epigenetic pathways [75,76,77,78]. Environmental exposures such as diet, stress, and toxins can impact epigenetic markers differently in males and females, contributing to sex-specific disease risks. For example, it was reported that the DNA methylation patterns of female and male children were differently impacted by lead exposure [79]. Moreover, the exposure to the endocrine disruptor bisphenol A (BPA) was also able to induce DNA methylation alteration in a sex-specific manner [80]. Also, it was reported that female and male mice subjected to stressful behavioral experiences, including a schedule of food rewards, or an episode of forced swimming followed by restraint stress, or no specific behavioral task, exhibited different changes in histone modifications [81]. For example, males showed higher levels of H3K27me3 in the frontal cortex, while females showed a reduction in H3K9ac, H3K4me3, H3K9me2, and H3k27me3 in the hippocampus. Similarly, sex-specific histone modifications were observed in brain samples from mice exposed to lead [82] and arsenic [83].

**Figure 2:**
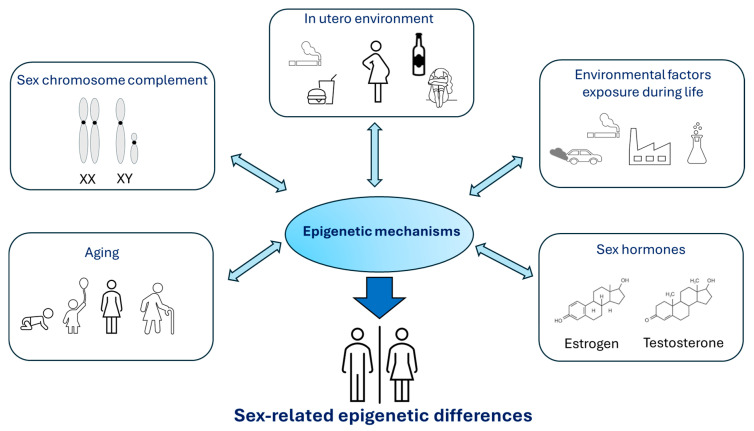
Overview of the main factors that contribute to sex-related epigenetic differences.

Overall, females and males exhibit different epigenetic marks, which could account for differences in an organism’s physiology and in its ability to respond to environmental factors, potentially predisposing to sex-related risks of human pathologies. Figure 2 provides an overview of the factors that contribute to sex-related epigenetic differences.

## 3. Epigenetic Mechanisms Underlying Sex Differences in Central Nervous System

The sexual differentiation of the brain begins prenatally and continues throughout life, shaped by the interplay of gonadal hormones and sex chromosomes. Moreover, environmental factors can influence central nervous system development, and there is evidence that females and males can respond differently to environmental factors, undergoing different effects on the brain [84,85]. Accumulating evidence highlights that epigenetic mechanisms play an important role in shaping sex-related neuronal differentiation.

Brain masculinization and feminization are modulated by estrogen receptor α (ER-α) and estrogen receptor β (ER-β) [86]. Specifically, ER-α primarily contributes to brain masculinization, while ER-β plays a major role in the defeminization of sexual behaviors [87]. The genes *Esr1* and *Esr2*, encoding for ER-α and ER-β, respectively, are regulated by the DNA methylation of their promoters. Notably, the methylation of *Esr1* and *Esr2*, under the influence of estradiol derived by the conversion of testosterone in neurons, differs between females and males during brain sexual differentiation [88,89]. The masculinization of neonatal females induced by the exposure to dihydrotestosterone and estradiol was accompanied by the downregulation of DNMT3a expression in the amygdala [90]. Perinatal testosterone exposure induced DNA methylation changes in adulthood, and methylation levels at a substantial number of sexually dimorphic CpG sites were masculinized [91]. A study conducted in male, female, and estradiol-treated masculinized female rat pups examined the activity and regulation of DNA methyltransferases (Dnmts) and the levels of DNA methylation in the development of the preoptic area (POA) [92]. The authors observed that males had lower Dnmt activity than female rats, that estradiol reduced Dnmt activity promoting the masculinization of POA, and that the maintenance of feminization required the maintenance of DNA methylation. This study suggested that brain feminization is not a passive process, but instead, brain feminization is maintained by the active suppression of masculinization via DNA methylation [92]. Interestingly, the authors observed that the deletion of *Dnmt3a* was sufficient to masculinize sexual behavior in female mice. Later investigations showed that males had a higher expression of *Tet* genes, which encode for enzymes involved in active DNA demethylation during neonatal life, and the testosterone treatment of females partially masculinized *Tet* expression [93]. On the other hand, *Dnmt* expression was similar between male and female rats, although females showed higher expression in POA on post-natal day 7 [93]. Moreover, by inhibiting DNA methylation, sex differences in the POA and bed nucleus of the stria terminalis (BNST) rat brain areas were eliminated [94]. It has also been observed that epigenetic mechanisms are important for the neuroendocrine control of puberty in females. A study performed in female rats showed that two genes, *Eed* and *Cbx7*, increased in the hypothalamus before puberty, and the inhibition of DNA methylation resulted in the failure of puberty [95]. So, DNA methylation changes mediate hormonal effects during puberty. In line with this, an investigation carried out in the blood of 325 individuals, including 140 females, at ages 10 and 18 years, showed that adolescent transition is associated with changes in DNA methylation levels at more than 15,000 CpG sites, the majority of which were associated with genes involved in the growth and development of the reproductive system [96]. Moreover, it has been observed that the chromatinic state in the female ventral hippocampus of mice fluctuated with the estrous cycle [97]. Particularly, changes in chromatin conformation occurred at the level of genes involved in neuronal function and behavior. Of note, a study performed in various central nervous system regions of mice, including hippocampus, cortex, cerebellum, brainstem, and retina, showed that components of the major histocompatibility complex I (MHCI) pathway changed during aging in a sex-specific manner, likely through epigenetic mechanisms [98]. Indeed, the expression of two genes encoding for MHCI receptors, namely, H2-K1 and H2-D1, undergoes CpG and non-CpG promoter methylation in the different brain regions investigated. Such DNA methylation changes correlated with age and differed between sexes showing a DNA methylation increase in female mice. It has also been reported that specific methylation levels of CpG and non-CpG sites in young and old mouse hippocampus undergo age-related increases or decreases that are predominantly sexually divergent, suggesting a regulation of brain DNA methylation with aging by sex [99]. Moreover, the authors also confirmed these results in humans by analyzing DNA methylome data from 19 hippocampal and 145 frontal cortexes of neurologically healthy individuals aged 13–95 [99].

Histone modifications have also been implicated in brain masculinization driven by hormones. For example, treatment with the HDAC inhibitor valproic acid counteracted the masculinization effect of testosterone in the mice BNST development [100]. It has also been observed that estradiol, derived from testosterone metabolized by aromatase, exerted its masculinizing effects on brain development through chromatin remodeling induced by its transcription factor estrogen receptor-α [101]. Moreover, the inhibition of histone acetylation in newborn male rats reduced male sexual behavior in adulthood [102]. These studies are only a few examples that suggest that chromatin remodeling is implicated in sexual dimorphism mediated by sexual hormones. Some of the epigenetic differences between females and males in brain physiology could be linked to XCI, and particularly to escape genes. Indeed, some of the escape genes encode for epigenetic regulators. For example, an escape gene is the *KDM5C* that encodes a histone demethylase whose depletion induces the upregulation of several genes, likely accounting for sex differences in intellectual disability [103,104]. Thus, the epigenetic differences between males and females could partially account for the sexual chromosomal constitution of the individuals.

Also, non-coding RNA expression in the brain shows sexual dimorphism [105]. An investigation performed in the hypothalamus of mice showed that neonatal miRNA patterns were different between females and males and were dynamically responsive to estrogen [106]. Particularly, 162 miRNAs with sex-biased expression were identified, 92 of which were estrogen-responsive. Moreover, deregulated miRNAs were involved in the expression of genes involved in the sexual differentiation of the brain, such as *Esr1*, *Esr2*, androgen receptor (*Ar*), and aromatase (*Cyp19a1*). Furthermore, many miRNAs exhibiting expression differences between males and females with neurodevelopmental roles have been detected in the embryonic mouse brain [107].

Epigenetic mechanisms have also been implied in the environmental-induced sex differences in brain characteristics. For example, male mice, but not female, exposed to chronic stress during gestation (such as exposure to constant light, exposure to fox odor, restraint stress, and multiple cage changes) showed maladaptive behavioral stress responsivity and depressive-like behavior, along with altered methylation and expression of two stress-related genes, namely, corticotropin-releasing factor (*Crf*) and glucocorticoid receptor (*Nr3c1*) in the hypothalamus and amygdala neurons [108]. Moreover, an increment in *Bdnf* gene methylation was detected in the hippocampus and peripheral blood of male mice but not in females exposed during pregnancy to BPA, along with neurobehavioral deficits [109]. The importance of epigenetic mechanisms in inducing sex-specific brain characteristics was also observed by the genome editing approach. The behavioral consequence of the epigenetic editing of the *Cdk5* gene, which consisted of the histone acetylation of its promoter in the hippocampus, was investigated in female and male mice [110]. Targeting the histone acetylation of hippocampal *Cdk5* promoter attenuated fear memory retrieval in female but not in male mice, suggesting a sex-specific mechanism underlying *Cdk5* expression. Moreover, by overexpressing the long non-coding RNA *FEDORA*, in both neurons and oligodendrocytes of the prefrontal cortex, depression-like behavior abnormalities were induced in female mice but not in males, thus further corroborating the important role mediated by epigenetic mechanisms in sex-specific brain physiology [111]. These epigenome-editing studies suggest that the epigenetic modifications observed in males and females may drive brain alterations rather than merely being a consequence of them.

Such differences in sex-related epigenetic patterns of the central nervous system could account for differences in susceptibility, clinical presentation, and progression of neurological diseases in females and males. A study conducted in 1408 post-mortem brain samples identified thousands of differentially methylated CpG sites among females and males [112]. By analyzing the regulatory networks associated with the differentially methylated genes, it was observed that the majority of them are involved in psychiatric disorders, such as *NRXN1*, *NRXN2*, *NRXN3*, *FDE4A*, and *SHANK2*, thus suggesting that sex differential DNA methylation may underlie the sex-bias of these diseases. A whole-genome bisulfite sequencing was performed in the human dorsolateral prefrontal cortex from 20 prenatal samples during the second trimester in utero [44]. Authors found that the majority of CpG sites were methylated and increased with age, and that males tended to have higher methylation compared to females. The autosomal sex-related CpG sites were associated with genes involved in synaptic transmission, regulation of GTPase activity, and the glutamate receptor signaling pathway, and they were particularly enriched for autism spectrum disorder-associated genes. Also, the downregulation of a lncRNA named *LINC00473*, which is involved in neuronal function, was reported in the prefrontal cortex of women diagnosed with depression, while men with the same diagnosis showed *LINC00473* expression levels comparable to those of male control subjects [111].

## 4. Epigenetic Differences Between Sexes in Neurodegenerative Diseases

### 4.1. Sex-Related Epigenetic Alterations in Alzheimer’s Disease

From a neuropathological point of view, Alzheimer’s disease (AD) is characterized by the extracellular accumulation of the amyloid β (Aβ) peptides, resulting from the processing of amyloid precursor protein (APP) and the presence of intraneuronal neurofibrillary tangles derived by the hyperphosphorylation of tau protein. In the predominant pathway, APP undergoes sequential cleavage by α-secretase, followed by γ-secretase, after which no toxic Aβ peptides are produced. In contrast, in the amyloidogenic pathway, APP is first cleaved by β-secretase, producing a soluble fragment that is then processed by γ-secretase to release the Aβ peptide [113]. Early-onset familial forms of AD (EOAD, onset before 65) derive from causative mutations in specific genes. Until now, pathogenic mutations in three genes, namely, *APP,* and *PSEN1*, and *PSEN2* (which encode for subunits of γ-secretase), are frequently identified in EOAD patients. However, EOAD represents only a minority of AD cases, and most AD patients (>95% of cases) are the result of an interplay between predisposing genetic variants and environmental risk factors. In these sporadic forms of AD, the onset typically occurs after the age of 65 (LOAD, late-onset AD). In LOAD, several genetic variants have been associated with an increased risk of developing the disease [114]. However, the best characterized genetic variants associated with LOAD are the polymorphisms of the apolipoprotein E (*APOE*) gene. Particularly, there exist three isoforms of *APOE*, namely, *APOEε2*, *APOEε3*, and *APOEε4*. The *APOEε4* allele increases the risk of developing AD, while the *APOEε2* allele has been associated with decreased risk [115]. Interestingly, the *APOE* genotype exerts different effects on males and females in the risk of AD. Indeed, the *APOE*ε4 confers a greater risk of developing the disease in women than men, and a recent meta-analysis including 27 independent research studies and nearly 58,000 participants revealed that this difference is particularly relevant in individuals aged 65–75 years and that women between ages 55–70 years had a higher risk of developing mild cognitive impairment, the prodromal stage of AD [116,117]. It has been suggested that the heightened risk in women may result from interactions between *APOE* and estrogen [118]. Several studies are pointing to the role of epigenetic mechanisms in AD, showing their potential role in the onset and progression of the disease and their potential use as peripheral biomarkers [119]. However, only a few studies have aimed at searching for specific epigenetic alterations in female and male subjects affected by AD. Following in this section is reported the main evidence of sex-specific epigenetic alterations in AD pathogenesis (Table 1).

In a study performed in 2006, the methylation levels of the *APP* promoter were analyzed in the cerebral cortex of adult and old mice part, of which were treated with sex hormones, including testosterone and estradiol, or subjected to gonadectomy [120]. The authors observed that *APP* promoter methylation increased in females and was sensitive to sex hormones. Particularly, testosterone decreased *APP* methylation in adult male and female mice and increased methylation in old male and female mice. Estradiol treatment increased *APP* methylation in adult and old male mice, while in females, it induced decreased methylation in adult mice and increased methylation in old mice. Moreover, a subsequent study identified hypomethylation of the Aurora Kinase C (*AURKC*) gene, which is involved in the mitosis process, in brain samples of AD patients compared to controls, but the difference was specific to male subjects [121]. By using publicly available DNA methylation data derived from various brain regions of healthy adult subjects and AD patients, some differentially methylated CpG sites between females and males have been observed [122]. However, the sex-dependent CpG sites were not significantly associated with AD. Two meta-analyses of genome-wide investigation studies performed in brain and blood, respectively, identified distinct sex-specific DNA methylation patterns related to AD [123,124]. In the meta-analysis performed by Zhang and collaborators, DNA methylation data at the genome-wide level from four independent cohorts were employed, including 1030 prefrontal cortex brain samples, of which 642 were from female subjects. The authors searched for associations between DNA methylation levels and the Braak stage, a widely used method to diagnose AD post-mortem, in females and males. Several CpG sites whose methylation correlated with the Braak stage in a sex-specific manner were identified. Gene ontology and pathway analysis identified the biological processes enriched with significant methylation changes in males and females. One of the top biological processes for females was the response to platelet-derived growth factor, while in males the dysregulation of the complement system. Of note, 14 identified CpG sites were not identified in previous genome-wide investigations, suggesting the importance of considering the sex of individuals to identify epigenetic alterations [123]. In a later meta-analysis, peripheral blood methylome data were obtained from two independent cohorts including 632 females (188 AD and 444 controls) and 652 males (239 AD and 413 controls). The altered methylation patterns of AD differed in females and males, revealing that sex-specific epigenetic schemes underlying AD are detectable also in peripheral tissues [124].

Also, sex-related differences in histone modifications have been identified in AD. Casciaro et al. investigated in the brain cortex of an AD mouse model (PSAPP) the genome-wide profiles of the histone marks H3K4me3, H3K27ac, and H3K27me3 [125]. The global distribution of H3K4me3 and H3K27me3 revealed alterations on the X chromosome that could distinguish between males and females, whereas the distribution of H3K27ac was more uniform across sexes. Histone modifications have also been investigated in the dorsal hippocampus of the senescence-accelerated mouse prone 8 (SAMP8), which is an animal model of late-onset AD, and of its mouse control (senescence-accelerated mouse resistant, SAMR1) characterized by normal aging [126]. The authors found a general reduction of H3K27me3 in SAMP8 mice and a specific reduction of histone 3 acetylation in SAMP8 male mice. Moreover, histone modifications occurred with an increase in histone deacetylase (HDAC2) and the mRNA of the histone demethylase Jumonji domain-containing protein-3 (*Jmjd3*) in SAMP8 mice compared to SAMR1. On the other hand, HDAC4 and Dnmt3a levels were lower in both male and female SAMP8 mice compared to SAMR1, while females exhibited a decrease in HDAC1 and an increase in *Utx*, the gene encoding a lysine demethylase [126].

Regarding non-coding RNA, altered sex-related lncRNAs expression was detected between males and females in the superior frontal gyrus of AD and control subjects [127]. Particularly, the authors found 13 sex-associated lncRNAs that were dysregulated in AD brains compared with normal brains. Moreover, the expression levels of the sex-associated lncRNAs *RNF144A-AS1*, *LY86-AS1*, and *LINC00639* showed a negative correlation with the Braak stage of the patients. In a following study, linear and circular RNA variants of *HOMER1* were found to be downregulated in the entorhinal cortex of female patients with AD compared to female control subjects, while no changes were observed in male individuals [128]. *HOMER1* encodes for a protein involved in synaptic function and Aβ toxicity in the initial phases of AD [129], and levels of circular RNA derived by *HOMER1* were previously found to be deregulated in AD brains [130]. In an attempt to unravel the role of vascular dysfunction in AD, the expression of miRNAs involved in vascular function and the regulation of amyloidosis was analyzed in brain vessel segments of AD mice [131]. The authors identified at least 20 cerebrovascular miRNAs dysregulated during the progression of the pathology, the majority of which were specifically altered in females or males. A recent meta-analysis was performed on six studies of microRNA expression in AD patients to identify sex-related alterations in miRNA expression [132]. Meta-analyses of miRNA expression profiles in blood samples revealed the alteration of 16 miRNAs in female and 22 miRNAs in male AD patients. Functional enrichment analysis revealed that the most affected processes were related to activities of kinase enzymes and apoptosis in males, and RNA metabolism in females. On the other hand, brain samples meta-analysis showed the dysregulation of six miRNAs in females and four miRNAs in males, and no sex-related biological process to those miRNAs was identified.

**Table 1:** Sex-related epigenetic alterations in Alzheimer’s disease studies.

Experimental Model	Epigenetic Target	Observation	Reference
Cerebral cortex of female and male mice	*APP* gene methylation	*APP* promoter methylation was higher in female mice	[120]
Inferior temporal gyrus samples from 30 AD and 30 controls (15 females and 15 males per group)	DNA methylation at the genome-wide level and pyrosequencing of *BRCA1*, *ZNF714*, and *AURKC* genes	Genome-wide analysis revealed hypomethylation of *BRCA1* and AURKC and hypermethylation of *ZNF714* in AD. Pyrosequencing analysis showed that *AURKC* hypomethylation was specific to male subjects	[121]
DNA methylome data from temporal, frontal, entorhinal cortex, and cerebellum derived by 15 datasets, including AD and control subjects	DNA methylation at the genome-wide level	DNA methylation differences between males and females were homogeneous in the four brain regions. Sex-related CpG sites were not associated with AD condition	[122]
Prefrontal cortex of 1030 individuals (642 females, 388 males)	DNA methylation at the genome-wide level	Already known AD-related genes neuropathology, such as *MBP* and *AZU1*, had sex-specificity. Moreover, fourteen new differentially methylated positions located in *TMEM39A* and *TNXB* were found	[123]
Peripheral blood of 632 females (188 AD and 444 controls) and 652 males (239 AD and 413 controls)	DNA methylation at the genome-wide level	Several sex-specific CpG methylation differences between AD and control subjects were identified. The most associated CpG sites with AD mapped to *PRRC2A* and *RPS8* genes and were significant only in females	[124]
Brain cortexes of 5 female and 5 male mice expressing the mutated human *APP* and *PSEN1* genes (PSAPP mice)	Genome-wide profiles of H3K4me3, H3K27ac, and H3K27me3	Sex differences were observed for X-chromosome H3K4me3 and H3K27me3	[125]
Dorsal hippocampus of female and male AD mice (senescence-accelerated mouse prone 8, SAMP8) and control mice (senescence-accelerated mouse resistant, SAMR1)	Global H3Ac, H4Ac, and H3K27me3. Gene expression of histone demethylases (including *Jmjd3* and *Utx*) and methylases	Global levels of H3Ac were decreased in male AD mice compared to male control mice. Gene expression of *Utx* and *Jmjd3* were increased in AD and control females compared to AD and control males.	[126]
Expression profile of brain tissues derived by microarray datasets, including 27 females (13 AD and 14 controls) and 16 males (7 AD and 9 controls)	LncRNA expression at the genome-wide level	Expression levels of 13 sex-associated lncRNAs were dysregulated in AD brains. Three of them, including lncRNAs *RNF144A-AS1*, *LY86-AS1*, and *LINC00639*, decreased with the severity of the disease	[127]
Entorhinal cortex of 28 AD cases (18 females, 10 males) and 16 controls (6 females, 10 males)	Three variants of lncRNA *HOMER1*	Two *HOMER1* variants were downregulated in female cases with respect to control cases. No differences were detected in male subjects	[128]
Brain vessel segments of 3xTg-AD mice chategorzied in 4 groups (3 females and 3 males per group) based on their disease stage: young, cognitive impairment, presence of extracellular Aβ, and presence of Aβ and neurofibrillary tangles	Expression of 599 miRNAs involved in vascular function and regulation of amyloidosis	Downregulated expression of twenty miRNAs indictaed transition from pre-AD to AD pathology. Ten of these miRNA were specifically altered during disease progression in males (miR-126-3p, miR-150, miR-151-5p, miR-23a, miR-34b-3p; let-7d, let-7i, miR-132, miR-181a, and miR-99a), and two in females (miR-150 and miR-539).	[131]
Meta-analysis of six miRNA expression studies in peripheral blood (two studies) and brain samples of AD patients (four studies)	miRNA expression at the genome-wide level	Peripheral blood alteration of 16 miRNAs in female (all miRNAs overexpressed) and 22 miRNAs in male (18 overesxpressed, 4 underexpressed) AD patients. Brain analysis showed that five miRNAs were underexpressed (miR-767-5p, miR-668, miR-494, miR-653, miR-431-3p) and 1 miRNA overexpressed (miR-105-3p) in AD females compared to control females; regarding males, 2 miRNAs were overexpressed (miR-491-3p and miR-3149) and 2 miRNAs underexpressed (miR-767-5p and miR-7-5p) in AD patients compared to controls.	[132]

### 4.2. Sex-Related Epigenetic Alterations in Parkinson’s Disease

Parkinson’s disease (PD), the second most prevalent neurodegenerative disorder after AD, is clinically defined by motor symptoms, including resting tremors and bradykinesia, as well as non-motor symptoms like cognitive impairment and dementia [133]. The brains of PD patients are marked by dopaminergic neuron death in the substantia nigra, along with the formation of intracytoplasmic inclusions known as Lewy bodies containing aggregates of α-synuclein. Most cases of PD are sporadic, with Mendelian forms accounting for only 5–15% of cases. The *SNCA* gene, which encodes the α-synuclein, was the first gene identified as causing autosomal dominant PD. To date, in addition to *SNCA* over 20 genes, such as *LRRK2*, *CHCHD2*, *PRKN,* and *PINK1*, have been associated with monogenic forms of PD [134]. Several common genetic variants with small effect sizes have been associated with sporadic forms of PD [135]. Interestingly, a recent GWAS study in the Korean population identified a polymorphism of the *LRRK2* gene that was associated with an increased risk of PD in females but not in males [136]. Otherwise, a polymorphism of the *PARK16* gene was associated with an increased risk of PD only in male subjects. Several reports highlight that epigenetic mechanisms may play a pivotal role in the etiology of PD, although the role of sex has been often overlooked [137]. Following is reported some evidence that epigenetic mechanisms are altered in PD in a sex-specific manner (summarized in Table 2).

By investigating the epigenetic effects of PD induced by treatment with the pesticide dieldrin, sex-related CpGs methylation alterations were identified in the substantia nigra of treated mice [77]. DNA methylation alterations were observed in several genes related to dopaminergic neuron development and maintenance, including *Nr4a2* and *Lmx1b* genes. In a later study, it was observed that such sex-specific DNA methylation changes were detectable at multiple time points, from birth to adulthood [78]. These findings suggest that males and females exhibit distinct epigenetic responses to PD-related exposures. Additionally, neurodevelopmental exposure may trigger epigenetic changes that persist beyond the exposure period, affecting critical pathways and contributing to the development of PD in adulthood. The same research group investigated the genome-wide DNA methylation levels of the parietal cortex sampled from PD and control subjects [138]. This study showed that DNA methylation changes in PD brain samples differed between sexes. Some of the genes identified are known to be associated with PD, such as *DJ-1*, *VGLUT2*, *IA-2β*, and *NURR1*, but not-previously PD-related genes involved in neurodevelopment and neurotransmission with sex-specific methylation patterns were also identified.

In addition to sex-related DNA methylation alterations, differential ncRNA expression in females and males has also been detected in PD patients. In a study performed in dopamine neurons of PD and control subjects, it was observed that miRNAs were dysregulated [139]. Their expression differed between females and males, showing a tendency for a higher number of upregulated miRNAs in males, the majority of which were involved in synaptic transmission and apoptosis. In a subsequent study, the expression profiles of the microRNA29 family (miR-29s) were evaluated in blood serum from patients with PD and healthy controls [140]. It was found that serum miR-29s levels were significantly downregulated in PD patients compared to control subjects and that they were markedly higher in females compared to male PD patients. Another study evaluated the expression levels of *miR-132*, which is known to be altered in the brain of PD animal models, in plasma samples of PD patients, control subjects, and individuals with various non-PD neurological disorders (NDCs) [141]. Considering both sexes, the *miR-132* expression levels were significantly higher in PD than in NDC and control subjects. When considering the sex of the subjects, PD had higher plasma levels of *miR-132* compared to the control subjects, but not with NDC in male subjects, while no significant difference was evident in females. In a more recent study, the expression of specific miRNAs was evaluated in serum samples of female and male PD subjects [142]. The authors observed that one miRNA involved in neurogenesis and neuronal death, the *miR-34a-5p*, increased in PD male patients and decreased in females, while the *miR-146a-5p*, which is involved in inflammatory responses, increased in females and decreased in males. Also, disease duration significantly correlated with the expression of *miR-146a-5p*. Moreover, the expression of a lncRNA, named *LINC01259*, was downregulated in the peripheral blood of male PD subjects when compared to control males, and no differences in female subjects were observed [143].

### 4.3. Sex-Related Epigenetic Alterations in Amyotrophic Lateral Sclerosis

Amyotrophic lateral sclerosis (ALS) is a fatal motor neuron disease, in which motor neurons in the brain and spinal cord degenerate progressively. Genetic factors play a crucial role in ALS, with around 10–15% of cases classified as familial ALS. The remaining cases, known as sporadic ALS, do not have a clear link to inherited genetic mutations. However, common variants in known familial ALS-related genes, such as *SOD1*, *FUS*, *TARDBP*, and *C9ORF72*, can also be identified in sporadic cases [144]. In recent years, growing evidence has highlighted the involvement of epigenetic mechanisms in the etiology of ALS [145]. However, as with AD and PD, the role of sex in ALS-related epigenetic alterations has been rarely explored (summarized in Table 3).

A recent study that evaluated DNA methylation levels to measure the epigenetic age acceleration in the peripheral blood of ALS patients and control subjects considered the effect of sex [146]. The authors found that the epigenetic age acceleration associated with ALS status occurred independently of the sex of the subjects. However, a significant association between age acceleration and survival was detected in males but not in females [146]. A recent pre-print article showing a blood-based epigenome-wide association study meta-analysis in 9274 individuals (5529 males and 3975 females) including ALS and control subjects has been published [147]. A total of 226 ALS sex-associated CpG sites located in more than 150 genes were identified. Moreover, two ALS sex-associated differentially methylated positions (cg14380013 and cg06729676), which were significantly associated with survival, were detected. Specifically, cg14380013 is located in the *UNC119B* gene, which encodes for a transport factor that plays a role in the pathogenesis of ALS, and cg06729676 is located in the *NF1* gene [147]. These studies highlight that DNA methylation changes in ALS could be sex-specific, and some of them could also provide information on the estimated survival of the patients.

There has also been reported deregulation of ncRNA expression between female and male ALS patients. The expression levels of eleven miRNAs, selected based on previous investigations conducted in skeletal muscle and plasma samples from an ALS mouse model, were analyzed in serum samples from human ALS patients and control subjects [148]. It was found that the expression of two miRNAs, *miR-206* and *miR-106b*, was increased in ALS patients compared to controls. In the sex-stratified analysis, the authors observed that female patients had significant upregulation of *miR-206*, *miR-133b*, and *miR-145* compared to control females. On the other hand, male patients showed higher expression of *mi-R206* compared to control males, although the difference was not statistically significant. In a subsequent study, the expression levels of nine miRNAs involved in the regulation of muscle cells and inflammatory/angiogenic processes were investigated in the muscle tissue of 13 sporadic ALS patients [149]. Except for 1 miRNA, the other 12 miRNAs investigated were overexpressed in ALS patients compared to controls and in male patients compared to females. More recently, a study performed in the prefrontal cortex of 51 sporadic ALS and 50 control individuals showed that male ALS patients were characterized by a more profound deregulated expression of miRNA compared to females [150]. Indeed, 17 differentially expressed miRNAs were detected in males, while 9 in females. Moreover, males showed higher deregulation of hairpin miRNAs, which are the precursors of mature miRNAs, compared to females, thus suggesting potential defects in miRNA biogenesis as an early disease mechanism. The authors also highlighted that the mitogen-activated protein kinase (MAPK) pathway is altered early and demonstrated that trametinib, a MAPK inhibitor, has potential therapeutic benefits in vitro and in vivo, particularly in females [150].

## 5. Conclusions

In the present review, an overview of the main findings regarding sex-related epigenetic modifications in neurodegenerative diseases is reported. Epigenetic mechanisms are involved in human diseases, and an increasing number of epigenetic biomarkers are being identified for diagnosis, prognosis, and the discovery of therapeutic targets across a wide range of human pathologies, including neurodegenerative diseases [151]. Given the substantial differences in age of onset, prognosis, and duration of neurodegenerative diseases between females and males, the identification of sex-specific biomarkers is essential. In this context, epigenetic biomarkers could provide valuable insights, as they play a role in sexual differentiation and may potentially contribute to the sex-dependent development of these diseases. Epigenetic biomarkers change in a sex-dependent manner during aging, which is the main risk factor for neurodegenerative diseases. Further, evidence of sex-specific environmental effects on epigenome has been reported, and it is well known that environmental factors play a central role in the etiology of neurodegenerative diseases [152]. A better understanding of the sex-related epigenetic alterations could provide insights into molecular pathways relevant to clinical presentation and disease courses specific for females and males. Moreover, new sex-specific biomarkers for these conditions could be identified, improving diagnostic accuracy and prognostic evaluations. This understanding could guide the creation of therapies tailored to each sex, potentially improving treatment outcomes. Indeed, sex-specific analyses are infrequently conducted during the clinical trial phases for neurodegenerative diseases, and the significance of recognizing sex as a key factor influencing patient responses to treatments has largely been overlooked [153]. The difficulty in identifying new therapeutic strategies for the treatment of these diseases could be biased by the effects of sex, which could act as a confounding factor in the evaluation of the results. Moreover, it is also known that treatments for neurodegenerative diseases could have beneficial effects only in females or in males, so the mechanisms underlying this sex discrepancy need to be evaluated [154,155,156].

Given the growing importance of precision medicine in the treatment of human diseases, a deeper understanding of sex-specific epigenetic mechanisms is highly relevant for neurodegenerative diseases. The studies reported in the current review highlight the presence of sex-related epigenetic modifications in neurodegenerative diseases. However, the number of studies investigating this issue remains limited, making it difficult to draw definitive conclusions about their potential use in clinical practices. There are also significant limitations in the studies performed until now, particularly concerning the sample sizes of patient cohorts, which, in some cases, compromise the reliability of the findings. Future studies aimed at investigating the sex differences in epigenetic patterns should be designed by including an adequate sample size to enable analyses with sufficient statistical power. Moreover, longitudinal studies are needed to evaluate if sex-related epigenetic differences are a consequence of the disease or emerge before the symptoms.

Another limitation of the current literature is that epigenetic mechanisms are evaluated individually across different studies, despite their interactions with one another. For instance, lncRNAs regulate chromatin architecture by recruiting writers and erasers of DNA methylation and histone modifications [21]. Similarly, the methylation of genes encoding ncRNAs, along with the modification of their associated histones, can influence the expression of miRNAs and lncRNAs [157,158,159]. Given the sex-specific nature of epigenetic mechanisms, it is plausible that their interactions may also differ between females and males, leading to distinct effects in the central nervous system. A deeper understanding of these sex-specific interactions is, therefore, essential to better elucidate the role of sex-related epigenetic mechanisms in neurodegenerative diseases.

In conclusion, this review underscores the importance of considering sex differences in the investigation of epigenetic alterations associated with neurodegenerative diseases. A more in-depth understanding of these differences will inevitably improve our comprehension of the etiology of neurodegenerative diseases, enabling the identification of more precise biomarkers with clinical utility for diagnosis and prognosis, as well as the discovery of new therapeutic targets tailored to each individual.

## Figures and Tables

**Table 2:** Sex-related epigenetic alterations in Parkinson’s disease studies.

Experimental Model	Epigenetic Target	Observation	Reference
Substantia nigra of C57BL/6 mice exposed to the PD-linked pesticide dieldrin	DNA methylation at the genome-wide level	Dieldrin treatment induced sex-specific DNA methylation alterations in several genes related to dopaminergic neuron development and maintenance, including *Nr4a2* and *Lmx1b* genes, at multiple time points	[77,78]
Post-mortem parietal cortex of 50 PD (17 females, 33 males) and 50 (20 females, 30 males) control subjects	DNA methylation at the genome-wide level	Specific DNA methylation alterations in PD patients compared to controls were identified in male (including hypomethylation of *PARK7*) and female (including hypermethylation of *ATXN1*) subjects	[138]
Substantia nigra samples from 8 control subjects and 8 PD patients (3 females and 5 males per group)	Expression of 159 miRNAs	PD patients had dysregulated miRNA expression profiles (109 upregulated and 50 downregulated) with patterns of miRNA changes that show a trend of more upregulation in the male and more downregulation in the female group	[139]
Serum samples of 80 PD patients (32 females, 48 males) and 80 controls (32 females, 48 males)	MicroRNA29 family (*miR29* a, b and c) expression	*MiR-29* levels were decreased in PD patients. Expression of *miR-29a* and *miR-29c* was higher in females compared to males	[140]
Plasma of 269 PD patients, 176 neurological disease controls (NDC), and 222 healthy controls (HC), including 392 males and 275 females	*MiR-132* expression	*miR-132* expression was significantly higher in PD than in NDC and HC subjects. Higher plasma miR-132 levels in PD compared to HC were detectable in males but not in females.	[141]
Serum samples from 46 female and 58 male PD patients	*MiR-34a-5p, miR-146a, miR-155, miR-29a, and miR-106a* expression	MiR-34a-5p was increased in males, while miR-146a-5p was increased in females	[142]
Circulating leukocytes of 32 PD patients (15 females, 17 males) and 31 (12 females, 19 males) control subjects	Expression of the lncRNAs: *TTC3-AS1*, *LINC01259*, *ZMYND10-AS1*, *CHRM3-AS1*, *MYO16-AS1*, *AGBL5-IT1*, *HOTAIRM1*, *RABGAP1L-IT1*, *HLCS-IT1*, and *LINC00393*	LINC01259 expression was downregulated in male PD patients when compared to male control subjects	[143]

**Table 3:** Sex-related epigenetic alterations in amyotrophic lateral sclerosis studies.

Experimental Model	Epigenetic Target	Observation	Reference
Peripheral blood of 428 ALS patients (184 females, 244 males) and 288 control subjects (158 females, 130 males)	DNA methylation at the genome-wide level	Epigenetic age acceleration correlated with ALS status in both females and males and, only in males, with a shorter survival time	[146]
Peripheral blood from 6347 ALS and 2927 control subjects, including 3975 females and 5299 males	DNA methylation at the genome-wide level	226 CpG sites associated with ALS status sex-specifically, some of which were mapped in ALS genes such as *KCNQ1* and *HLA-F*	[147]
Serum samples from 12 ALS and 12 control subjects (6 females and 6 males per group)	Expression levels of 11 miRNAs: miR-133a, miR-206, miR-1, miR-145, miR-24, miR-19b, miR-17, miR-106b, miR-20a, miR-21, miR-133b	Increased expression of miR-206 and miR-106b in ALS patients versus controls. Sex-stratified analysis revealed that female patients had upregulation of miR-206, miR-133b, and miR-145	[148]
Muscle biopsies of 13 sporadic patients affected with ALS (6 males and 7 females) and 5 control subjects	Expression of muscle-specific miRNAs, including miR-1, miR-206, miR-133a, miR-133b, miR-27a, and inflammatory/angiogenic miRNAs, including miR-155, miR-146a, miR-221, and miR-149	Except miR-149, all miRNAs were overexpressed in ALS patients compared to controls and in male patients compared to females	[149]
Prefrontal cortex of 51 sporadic ALS (16 females, 35 males) and 50 control (28 females, 22 males) subjects	MiRNA expression at the genome wide level	Male ALS patients exhibited more significant downregulation in miRNA expression compared to females	[150]

## Data Availability

Not applicable.
